# KIRA6 restrains the generation of myeloid-derived suppressor cells and overcomes resistance to anti-PD-1 therapy

**DOI:** 10.1038/s41419-025-08401-6

**Published:** 2025-12-27

**Authors:** Chun Chen, Jing Chen, Xiaowen Lin, Jiali Hu, Yuncong Zhang, Dingjie Liu, Xumei Ouyang, Jing Li, Wenting Li, Shiying Xie, Ya Meng, Meixiao Zhan, Yongjun Peng, Hong-Wei Sun

**Affiliations:** 1 Guangdong Provincial Key Laboratory of Tumor Interventional Diagnosis and Treatment, Zhuhai Institute of Translational Medicine, Zhuhai People’s Hospital (The Affiliated Hospital of Beijing Institute of Technology, Zhuhai Clinical Medical College of Jinan University); School of Bioengineering, Zunyi Medical University, Zhuhai, China; 2 Department of Clinical Laboratory, The Affiliated Cancer Hospital of Zhengzhou University & Henan Cancer Hospital, Zhengzhou, China; 3 Guangdong Provincial Key Laboratory of Tumor Interventional Diagnosis and Treatment, Zhuhai Institute of Translational Medicine, Zhuhai People’s Hospital (The Affiliated Hospital of Beijing Institute of Technology, Zhuhai Clinical Medical College of Jinan University), Zhuhai, China; 4 The Department of Cerebrovascular Disease, Zhuhai People’s Hospital (The Affiliated Hospital of Beijing Institute of Technology, Zhuhai Clinical Medical College of Jinan University), Zhuhai, China; 5 Department of Pharmacy, The Fifth Affiliated Hospital of Zunyi Medical University, Zhuhai, China; 6 Guangzhou First People’s Hospital, the Second Affiliated Hospital, School of Medicine, South China University of Technology, Guangzhou, China; 7 Guangdong Provincial Key Laboratory of Tumor Interventional Diagnosis and Treatment, Department of Radiology, Zhuhai People’s Hospital (The Affiliated Hospital of Beijing Institute of Technology, Zhuhai Clinical Medical College of Jinan University), Zhuhai, China

**Keywords:** Cancer microenvironment, Preclinical research

## Abstract

Immune checkpoint blockade (ICB) therapy is one of the cornerstones of cancer treatment regimens, but the overall response rates remain low because of suppressive immune cells, such as myeloid-derived suppressor cells (MDSC). Therefore, it is unmet need to target MDSC to achieve better outcomes of ICB therapy. Inositol-requiring enzyme 1α (IRE1α) is identified as a key regulator for the generation of MDSC. Here, we evaluated the potential of KIRA6, an inhibitor for IREα kinase activity and RNase activity, to abrogate MDSC-mediated immune suppression. KIRA6 significantly suppressed 4T1 tumor growth, decreased MDSC population and enhanced T cell infiltration. Two dosages of KIRA6 treatment directly inhibited extramedullary myelopoiesis and MDSC generation in vivo. KIRA6 abrogated the induction of MDSC from bone marrow cells and abolished the immunosuppressive capability of MDSC in vitro. Meanwhile, KIRA6 not only attenuated G-CSF production from tumor cells thereby blocking the induction of MDSC, but also caused apoptosis of tumor cells. Moreover, KIRA6 treatment diminished MDSC generation, restored T cell proportion in both local and systemic immune landscapes and eventually overcame resistance to anti-PD-1 therapy. Our work establishes the evidence for KIRA6 as an impressive agent for abrogating MDSC-mediated immune suppression, killing tumor, and overcoming ICB resistance.

## Introduction

Immune checkpoint blockade (ICB) therapy has improved survival for cancer patients through harnessing the immune system, making it one of the cornerstones of cancer treatment regimens in a broad range of cancers nowadays [1–6]. However, the overall response rates remain low across many types of cancer, which limits the benefit of ICB therapy for majority of cancer patients [7–9]. Many immunosuppressive factors in the tumor microenvironment (TME) have been found to collaboratively restrain the activation and effector function of T cells despite the PD-1 blockade [10–12]. The generation of regulatory immune cells, such as myeloid-derived suppressor cells (MDSC), tumor-associated macrophages (TAMs) or regulatory T cells, is the major tumor-driven mechanisms of immune suppression, which alternatively impair antitumor T cell activity within the TME [10, 13–16]. Therefore, it is unmet need to target the suppressive immune cells in the TME to achieve better outcomes of ICB therapy.

Myeloid-derived suppressor cells (MDSC) constitute a major component of the TME, which are key orchestrators of immunosuppression in TME and could inhibit T cell function through multiple mechanisms, such as arginase-1 (ARG1) and reactive oxygen species (ROS) [17, 18]. The potent immune modulation capability of MDSC enables them to drive tumor progression, metastasis, and resistance to cancer therapies; therefore, MDSC infiltration is found to correlate with failure of ICB treatment and poor patient survival [18–22]. Considering their established role as therapeutic barriers, great efforts have been made to develop MDSC-targeting strategies for cancer therapy, including eliminating MDSC, blocking MDSC recruitment, and abrogating the suppressive function of MDSC [17, 23–26]. Although targeting MDSC in preclinical models get improved outcomes, there are still no drugs approved for clinical usage, urging for novel pharmacologic agents to dismantle this suppressive axis.

MDSC consists of a group of heterogeneous myeloid cells, including polymorphonuclear MDSC (PMN-MDSC) and monocytic MDSC (M-MDSC) [18]. It is generally believed that MDSC originate from hematopoietic progenitor cells in the bone marrow and spleen [18, 27–29]. This aberrant myelopoiesis process is driven by multiple cytokines and growth factors, such as granulocyte colony-stimulating factor (G‑CSF), promoting their differentiation into immunosuppressive effectors rather than functional counterparts [30–33]. Endoplasmic reticulum (ER) stress is observed in MDSC because of high secretory and metabolic activity during the immunosuppressive myelopoiesis in tumor milieu [34–37]. Inositol-requiring enzyme 1α (IRE1α), an important kinase and RNase of ER stress response, is identified as a key regulator for generation of MDSC [35]. On the one hand, IRE1α-mediated XBP1 cleavage promotes acquisition of suppressive activity of MDSC in cancer [35]. On the other hand, the kinase activity of IRE1α potentiates the expression of G-CSF and granulocyte/macrophage colony-stimulating factor (GM-CSF) from tumor cells through activation of JNK pathway, which further facilitates mobilization of hematopoietic progenitor cells and pathological myelopoiesis [38]. The dual effects of IRE1α make it an ideal target for abrogating MDSC generation.

KIRA6 (kinase-inhibiting RNase-attenuators), is developed for diabetes treatment by inhibiting IRE1α kinase activity and RNase activity simultaneously [39]. However, its potential for cancer treatment, especially for targeting MDSC generation and immunotherapy, remains unexplored. Here, we evaluate the antitumor activity of KIRA6 for breast cancer in both animal model and in vitro model. KIRA6 significantly suppresses 4T1 tumor progression and reprograms the tumor immune landscape by enhancing T cell infiltration and decreasing MDSC population. Short-term KIRA6 treatment and cell culture confirm that KIRA6 directly inhibits MDSC generation and function. Meanwhile, KIRA6 not only attenuates G-CSF production, therefore blocks the induction of MDSC, but also causes apoptosis in tumor cells. Moreover, KIRA6 treatment diminishes MDSC generation, restores T cell proportion in both local and systemic immune landscapes and eventually overcomes resistance to anti-PD-1 therapy. Our work establishes KIRA6 as an MDSC-targeting agent for overcoming ICB resistance and provides a promising combination strategy for achieving better immunotherapy.

## Materials and methods

### Reagents

Reagents used in this study are summarized in Supplementary Table S1.

### Tumor cell culture

4T1 cells (ATCC, CRL-2539) were cultured with RPMI 1640 medium supplemented with 10% fetal bovine serum (FBS), penicillin (100 U/mL), streptomycin (100 mg/mL) at 37 °C in 5% CO_2_-humidified atmosphere.

4T1 cells were plated overnight in complete RPMI 1640 medium before KIRA6 treatment. 4T1 cells were cultured with fresh culture medium with or without KIRA6 at indicated concentrations. For cell viability assay and flow cytometry analysis, cells were treated with vehicle or KIRA6 for 24 h. For the preparation of RNA and protein sample, cells were treated with KIRA6 for 4 h before sample collection. For tumor condition medium, 3 × 10^6^ 4T1 tumor cells were first plated in 10 cm dishes. When reached 80% confluence, tumor cells were treated with KIRA6 for 4 h and then washed and culture with 10 mL fresh medium for 24 h. Cell culture medium was collected and centrifuged to obtain tumor condition medium.

### Mouse model

All animal experiments were performed according to institutional guidelines and approved by the ethical board of Zhuhai People’s Hospital. Female BALB/c mice (6–8 weeks of age) were purchased from Bestest Biotechnology Company (Zhuhai, China). 2 × 10^5^ 4T1 cells were injected subcutaneously into the flank of BALB/c mice. Mice were intraperitonially injected with vehicles, KIRA6 (10 mg/kg) every two days or anti-PD-1 antibody (200 μg) every three days in designated groups. Tumor sizes were measured with caliper when tumors were palpable. Tumor volumes were calculated by (length × width^2^)/2.

### Isolation of leukocytes from bone marrow cells and splenocytes

Bone marrow cells were harvested by flushing the femurs and tibias of mice with PBS supplemented with 1% FBS using syringe. Splenocytes were obtained by homogenizing the spleen using nylon mesh and gently aspirating through a 21-gauge needle [28, 38]. Red blood cells were removed by ACK lysis buffer. Isolated cells were then washed and resuspended for cell culture, or flow cytometry analysis.

### Isolation of tumor-infiltrating immune cells

Mouse tumors were cut into small pieces and digested with 0.05% collagenase type IV, 0.002% DNase I in RPMI 1640 supplemented with 10% FBS. The dissociated cells were filtered through 100 μm mesh before washed and resuspended for FACS analysis or cell culture [33].

### Flow cytometry

Cells cultured in vitro, leukocytes from peripheral blood, spleen and tumor samples were prepared and suspended in PBS buffer supplemented with 1% heat-inactivated FBS, then stained with desired antibodies. Cell apoptosis was stained with APC Annexin V Apoptosis Detection Kit with 7-AAD. Cell cycle was detected with Cell Cycle and Apoptosis Analysis Kit. Data was acquired on DxP Athena (Cytek) and analyzed with FlowJo software.

### Generation of MDSC from bone marrow cells

Bone marrow cells were plated in 24-well plates in complete RPMI medium (with 10% FBS) supplemented with tumor condition medium in the presence of KIRA6. Cells were cultured at 37 °C in 5% CO_2_-humidified atmosphere for 3 days [38].

### Co-culture of MDSC and splenocytes

Splenocytes were isolated from homogenized spleen of naïve mice and stained with 1.5 μM CFSE for 10 min at 37 °C according to the manufacturer’s instructions. Then, splenocytes were co-cultured with induced MDSC at 2:1 ratio in the presence of 1 μg/ml anti-CD3 antibody, 1 μg/ml anti-CD28 antibody and 20 U/mL recombinant IL-2. Cells were cultured for 3–5 days and then collected, stained with surface markers, and analyzed by flow cytometry [29, 38, 40].

### Immunoblotting

The proteins were prepared with RIPA Lysis and Extraction Buffer and then quantified with BCA Protein Assay Kit. Proteins were separated by 10% SDS-PAGE, immunoblotted with anti-p-ERK, ERK, p-Myc, Myc, ARG1 and β-Actin antibody. Antibody binding was detected using horseradish peroxidase-conjugated anti-rabbit or anti-mouse IgG antibody and visualized with Western Chemiluminescent kit.

### RNA-seq and analysis

Cells were lysed with TRIzol for RNA purification. The library was constructed and then sequenced by Azenta on an Illumina instrument using a 2 × 150 paired-end (PE) configuration according to the manufacturer’s instructions. All data are publicly available in the GEO database (GSE304255). Differentially expressed genes were identified using DESeq2 (v1.34.0). Functional enrichment analyses were performed by Gene Set Enrichment Analysis (GSEA) or Gene Ontology (GO).

### G-CSF ELISA analysis

For analysis of G-CSF, tumor condition medium collected from vehicle or KIRA6-pretreated 4T1 cells were assessed for G-CSF concentration using the mouse G-CSF ELISA kits (MultiSciences) according to the manufacturer’s instructions.

### Statistical analysis

IBM SPSS Software (IBM Corporation) and GraphPad Prism (GraphPad Software) were used for statistical analysis. The significance of differences between groups was examined by the Student’s *t* test or Mann–Whitney test, as appropriate. The overall survival curves were generated by the Kaplan–Meier method and analyzed using the log-rank test. *P* < 0.05 was considered significant.

## Results

### KIRA6 inhibits tumor growth and modulates intratumoral immune cell infiltration

**Fig. 1:**
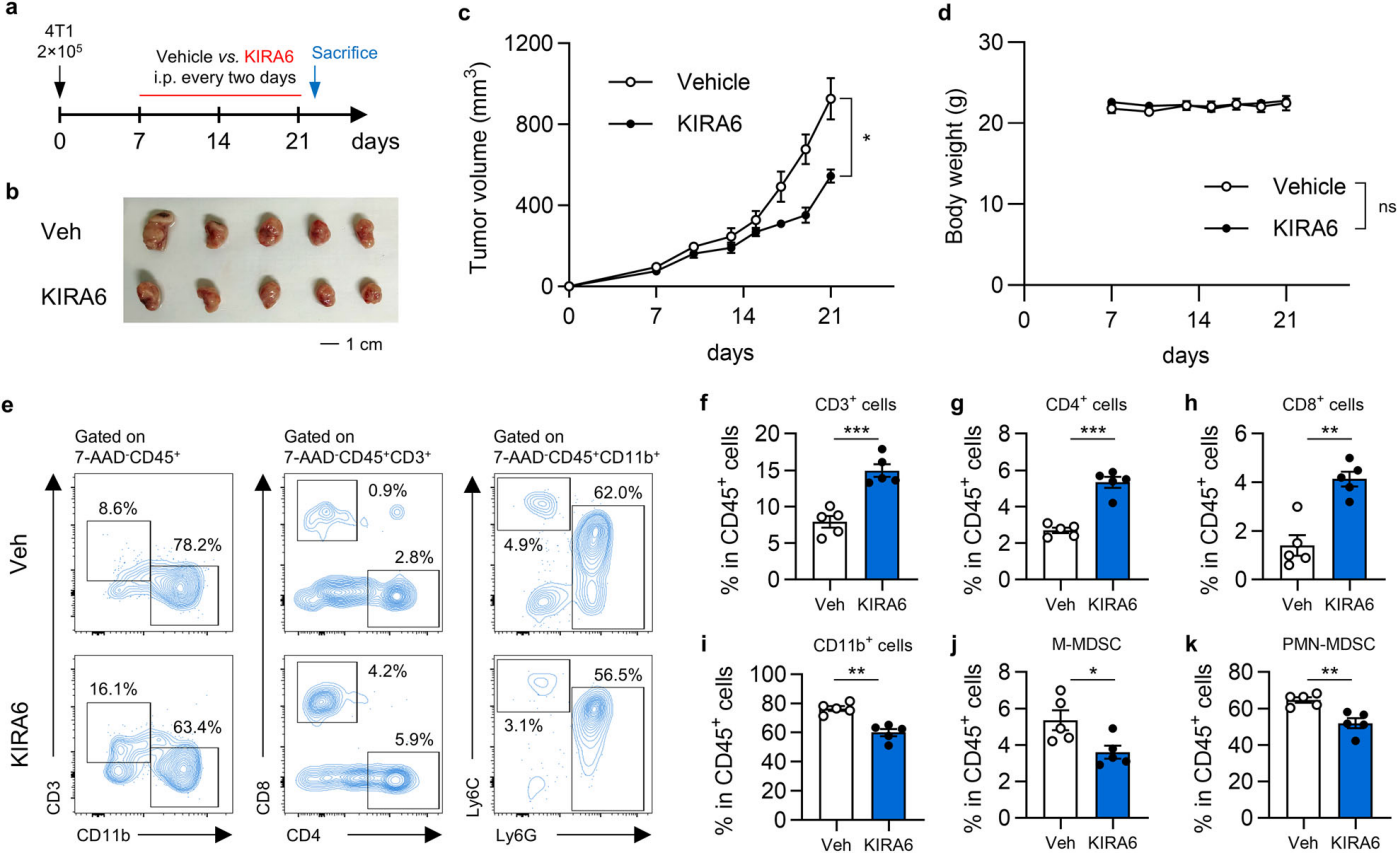
KIRA6 inhibits 4T1 tumor growth and modulates the tumor immune microenvironment. **a** Experimental treatment schedule. Female BALB/c mice with implanted 4T1 tumors were intraperitoneal (*i.p*.) injected with vehicle or KIRA6 every two days for two weeks. **b** Representative image of tumors from indicated groups at sacrifice. Scale bar,1 cm. **c** Tumor growth curves. Mean tumor volume of 4T1 tumors from vehicle or KIRA6-treated mice at the indicated time points. Results are shown as mean ± SEM. *n* = 5/group; *P < 0.05. **d** Body weight of vehicle or KIRA6-treated mice over the treatment period. Data is presented as mean ± SEM. *n* = 5/group; ns not significant. **e** Flow cytometry analysis of CD3^+^ T cells, CD11b^+^ myeloid cells, CD4^+^ T cells, CD8^+^ T cells, M-MDSC (CD11b^+^Ly6C^+^Ly6G^-^) and PMN-MDSC (CD11b^+^Ly6C^low^Ly6G^+^) in 7-AAD^-^CD45^+^ cells from 4T1 tumors treated with vehicle or KIRA6. The percentage of indicated cell populations in CD45^+^ cells is shown. **f**–**k** The frequency of the indicated immune cell subsets in total CD45^+^ cells from tumor tissues (*n* = 5 /group). The results are shown as mean ± SEM. *P < 0.05; **P < 0.01; ***P < 0.001.

To evaluate the antitumor efficacy of KIRA6, tumor-bearing mice were treated with KIRA6 in 4T1 tumor model (Fig. 1a). KIRA6 induced a significant reduction in tumor growth compared to vehicle-treated controls (Fig. 1b, c). Meanwhile, no significant changes in body weight were observed in between KIRA6 treatment mice and control mice (Fig. 1d), indicating favorable tolerability and absence of overt systemic toxicity at this dosage. Moreover, we further characterized the tumor immune microenvironment in KIRA6-treated mice via flow cytometry (Fig. 1e). KIRA6 significantly enhanced T cell infiltration in tumor tissues, with a marked increase in CD3⁺ T lymphocytes (Fig. 1f), CD4⁺ T helper cells (Fig. 1g), and CD8⁺ cytotoxic T cells (Fig. 1h) relative to vehicle-treated tumors. Conversely, KIRA6 injection markedly decreased CD11b⁺ myeloid cells (Fig. 1i), with specific reductions in both monocytic MDSC (M-MDSC, Fig. 1j) and polymorphonuclear MDSC (PMN-MDSC, Fig. 1k). Collectively, our data suggest that KIRA6 not only suppresses 4T1 tumor progression but also reprograms the tumor immune landscape by enhancing T cell infiltration and attenuating immunosuppressive myeloid populations.

### Short-term KIRA6 treatment reprograms systemic immunity

**Fig. 2:**
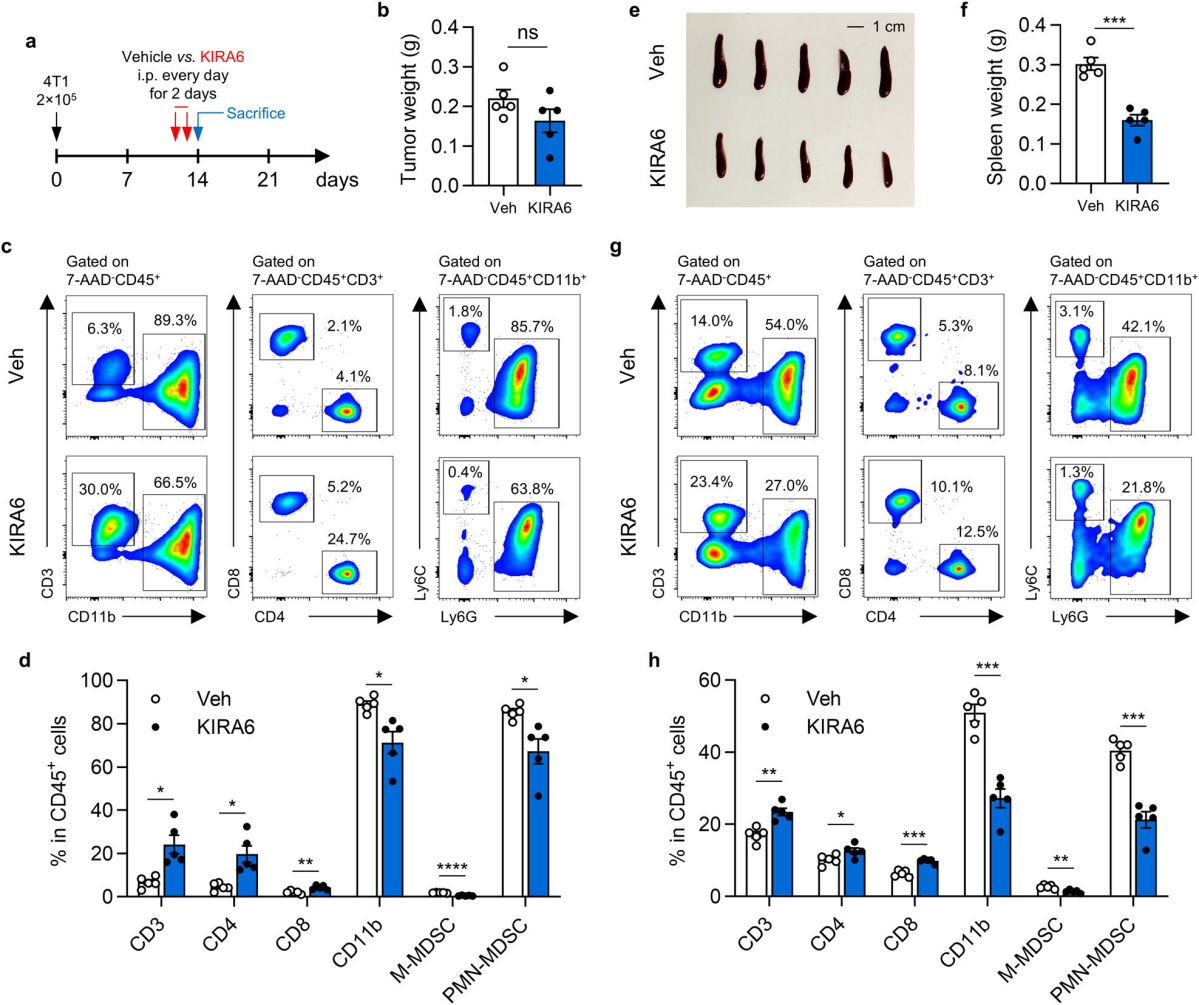
Short-term KIRA6 treatment reprograms systemic immunity. **a** Treatment schedule. 4T1 tumor-bearing mice received *i.p*. injections of KIRA6 or vehicle daily for two consecutive days and were sacrificed at day 14. **b** Tumor weight at sacrifice. Results are shown as mean ± SEM. *n* = 5/group; ns, not significant. **c** Flow cytometry analysis of immune cell subsets in 7-AAD^-^CD45^+^ cells from peripheral blood of 4T1 bearing mice treated with vehicle and KIRA6. The percentage of gated cell populations in CD45^+^ cells is shown. **d** The proportion of T cell subsets and myeloid populations in CD45^+^ cells from peripheral blood. The data is summarized as mean ± SEM. *n* = 5/group; *P < 0.05; **P < 0.01; ****P < 0.0001. **e** Image of spleens from indicated groups at sacrifice. Scale bar,1 cm. **f** Spleen weight at sacrifice. Results are shown as mean ± SEM. *n* = 5/group; ***P < 0.001. **g** Flow cytometry analysis of splenic immune cell subsets in 7-AAD^-^CD45^+^ cells from indicated groups. The percentage of gated cell subsets in total CD45^+^ cells is shown. **h** The proportion of splenic immune cell populations in CD45^+^ cells. The data is summarized as mean ± SEM. *n* = 5/group; *P < 0.05; **P < 0.01; ***P < 0.001.

To examine the direct immunological effects of KIRA6, tumor-bearing mice received short-term daily treatment for two consecutive days before sacrifice at day 14 (Fig. 2a). This brief intervention did not significantly reduce tumor weight compared to vehicle controls (Fig. 2b). Analysis of peripheral blood revealed that KIRA6 significantly increased the percentages of CD3⁺, CD4⁺, and CD8⁺ T cells within CD45⁺ leukocytes, while reducing CD11b⁺ cells, M-MDSC, and PMN-MDSC (Fig. 2c, d). In addition to circulating immune cells, we also examined the alteration in the spleen. KIRA6-treated mice exhibited marked reduction in spleen weight (Fig. 2e, f), suggesting potential effects on extramedullary hematopoiesis. In line with our observation in the peripheral blood, KIRA6 increased the proportions of CD3⁺, CD4⁺, and CD8⁺ T cells in CD45⁺ splenocytes, while decreasing CD11b⁺ cells, M-MDSC, and PMN-MDSC (Fig. 2g, h). These systemic immunomodulatory effects precede measurable tumor reduction, highlighting the direct immune reprogramming effect of KIRA6.

### KIRA6 directly inhibits MDSC differentiation and function in vitro

**Fig. 3:**
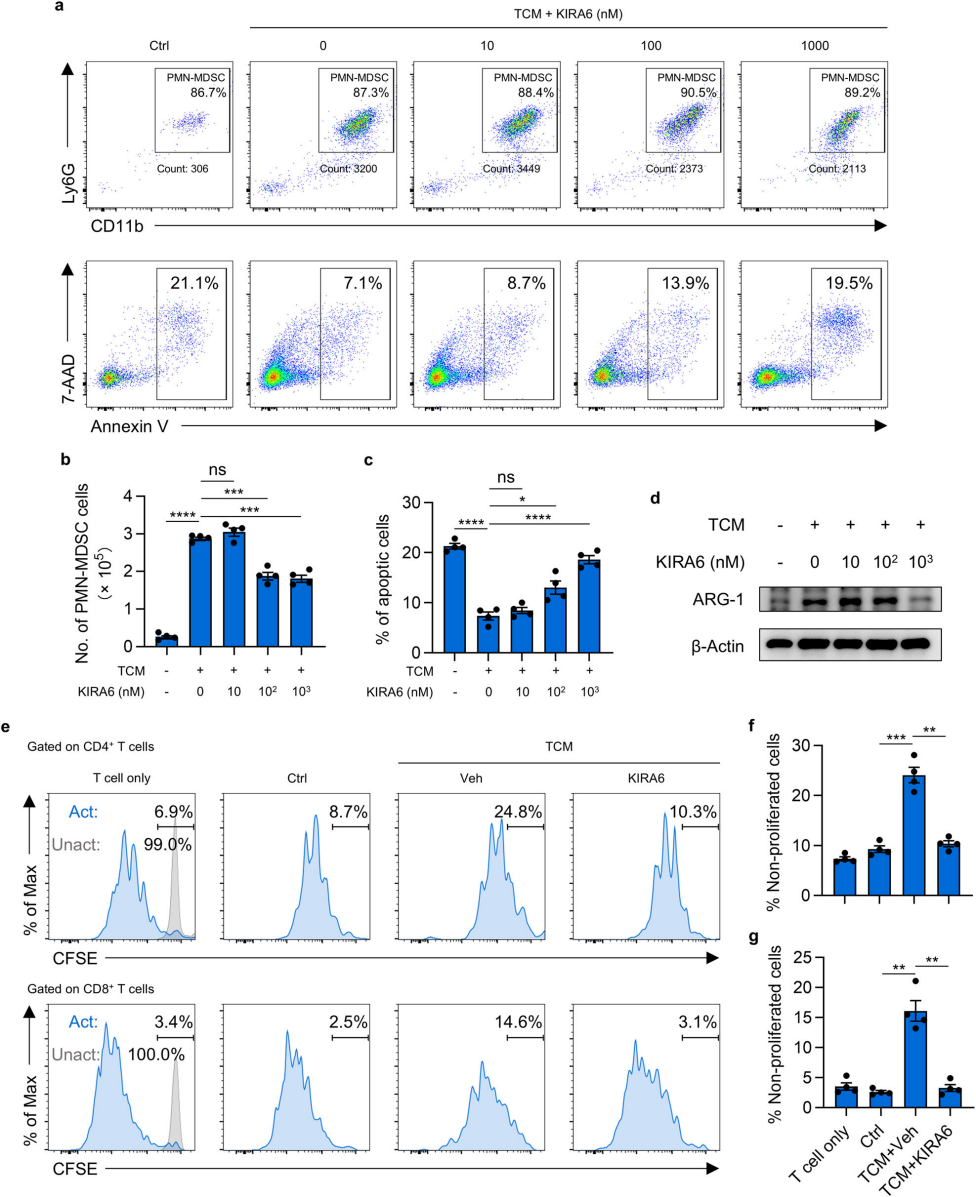
KIRA6 directly impairs MDSC generation and immunosuppressive function. **a** Bone marrow cells were cultured in control medium, 4T1 tumor-conditioned medium (TCM), or TCM with KIRA6 at indicated concentrations for 3 days. The quantification of PMN-MDSC and apoptosis of cultured cells were examined by flow cytometry. **b** The number of PMN-MDSC (CD11b^+^ Ly6G^+^) from indicated groups are summarized as mean ± SEM. *n* = 4/group; *P < 0.05; **P < 0.01; ****P < 0.0001. **c** The proportion of apoptotic cells is shown as mean ± SEM. *n* = 4/group; *P < 0.05; ****P < 0.0001. **d** Western blot analysis of ARG1 expression in MDSC cultured in (**a**). **e** CFSE-stained splenocytes were cultured alone or co-cultured with MDSC induced with control medium, 4T1 tumor-conditioned medium (TCM), or TCM with KIRA6 (1 μM) for 3 days (MDSC:T cell = 1:2). The proliferation of T cells was analyzed by flow cytometry. The proportion of non-proliferated CD4⁺ T cells (**f**) and CD8⁺ T cells (**g**) is shown as mean ± SEM (*n* = 4). **P < 0.01; ***P < 0.001.

To validate the direct effects of KIRA6 on MDSC, bone marrow cells from naïve BALB/c mice were cultured in 4T1 tumor-conditioned medium (TCM) in the presence of KIRA6 for 3 days. TCM potently induced PMN-MDSC differentiation, while KIRA6 suppressed TCM-induced MDSC in a dose-dependent manner (Fig. 3a, b and Supplementary Fig. S1). Meanwhile, KIRA6 treatment significantly induced PMN-MDSC apoptosis (Fig. 3a, c). Moreover, western blot analysis revealed KIRA6 markedly downregulated expression of the immunosuppressive enzyme arginase-1 (ARG1) in TCM-induced MDSC (Fig. 3d). To further assess functional consequences, TCM-induced MDSC were co-cultured with CFSE-labeled splenocytes. While TCM-MDSC profoundly suppressed T cell proliferation, KIRA6 treatment completely abolished their inhibitory capability on both CD4⁺ T cells and CD8⁺ T cells (Fig. 3e–g). In summary, our data suggest that KIRA6 directly inhibits MDSC generation and suppressive function in vitro.

### KIRA6 suppresses oncogenic signaling pathways

**Fig. 4:**
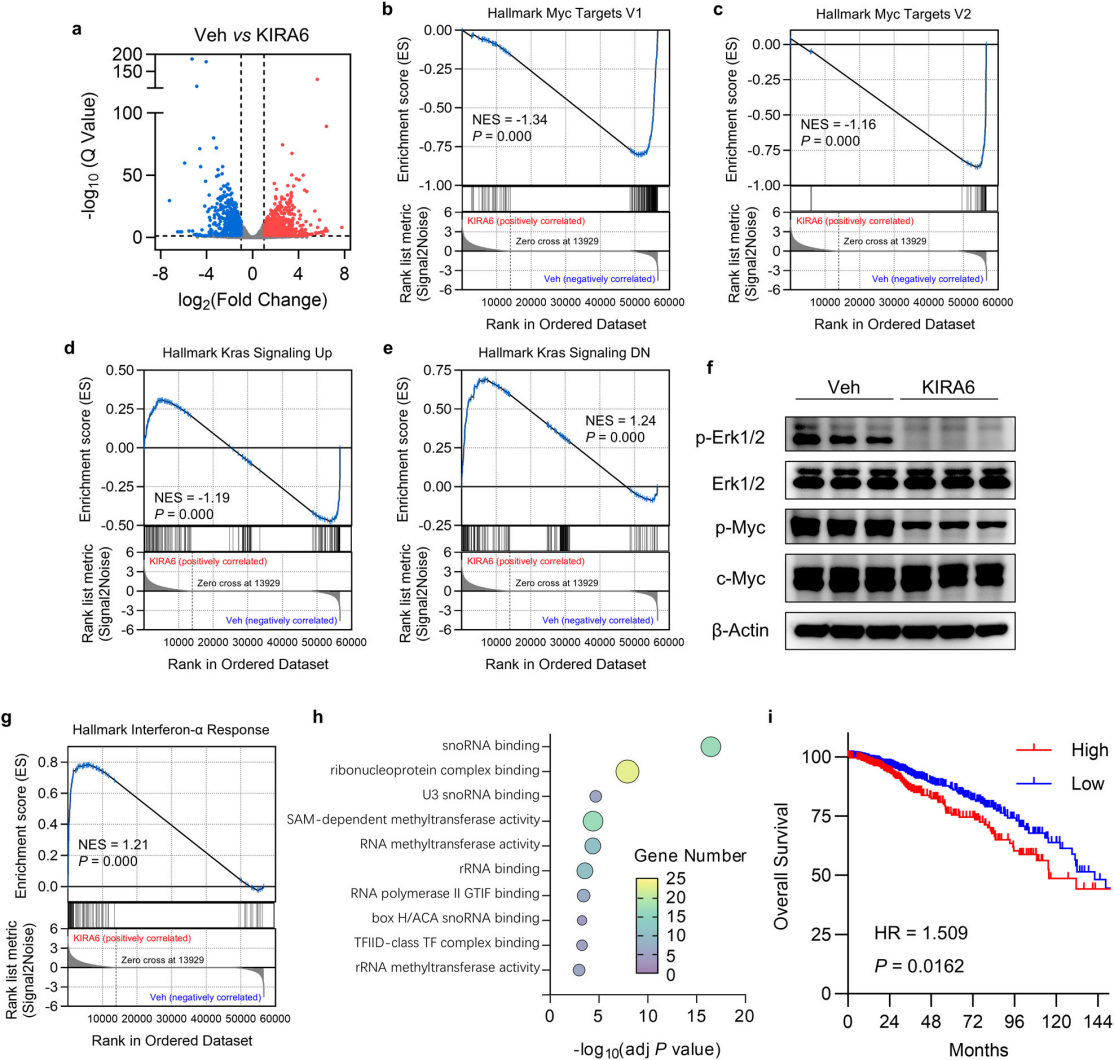
KIRA6 suppresses oncogenic signaling pathways. **a** Volcano plot of RNA-seq data from 4T1 cells treated with vehicle or KIRA6 (1 μM). **b**–**e** GSEA enrichment plot for Hallmark pathways enriched in vehicle- or KIRA6-treated 4T1 cells. NES, normalized enrichment score. **f** Western blot analysis of p-ERK1/2, total ERK1/2, p-Myc, and total Myc in vehicle and KIRA6-treated cells. β-actin was used as loading control. **g** GSEA enrichment plot for *Interferon-α Response* in vehicle and KIRA6-treated 4T1 cells. **h** Functional enrichment (GO Molecular Function) of KIRA6-downregulated genes. The top ten terms are shown. **i** Kaplan–Meier survival analysis for overall survival in TCGA breast cancer cohort (*n* = 1069). KIRA6-targeting gene signature was scored by Gene Set Variation Analysis (GSVA). Patients were stratified into high score group (*n* = 380) or low score group (*n* = 689) by the cutoff value (score = −0.4141) determined by receiver operating characteristic (ROC) curve. HR Hazard ratios.

We also investigated direct effects of KIRA6 on tumor cells by RNA sequencing. 4T1 cells were treated with KIRA6 for 4 h and then used for RNA sequencing (Fig. 4a). Gene set enrichment analysis (GSEA) was performed with RNA-seq data to reveal the alteration in key pathways. Hallmark pathways including *Myc Targets V1* (NES = −1.34), *Myc Targets V2* (NES = −1.16), and *Kras Signaling Up* (NES = −1.19) were significantly downregulated in KIRA6-treated cells (Fig. 4b–d). Conversely, KIRA6-treated cells exhibited enrichment of tumor-suppressive pathways, including *Kras Signaling Down* (NES = 1.24, Fig. 4e). These bio-informative analysis results indicated that KIRA6 significantly downregulated the Kras/ERK/Myc pathway, which is responsible for both G-CSF expression and cancer cell proliferation. Western blot further confirmed downregulation of phosphorylated ERK1/2 (p-ERK1/2) and phosphorylated Myc (p-Myc) proteins following KIRA6 treatment (Fig. 4f), indicating suppression of ERK/Myc signaling axes. Moreover, *Interferon-α Response* pathway (NES = 1.21) was significantly enriched in KIRA6-treated cells (Fig. 4g), suggesting that KIRA6 treatment induced an intrinsic antitumor immune response.

A list of genes (FPKM ≥ 5 in controls, log_2_FC <−1, *p* < 0.05) was identified from the RNA-seq data as core KIRA6-targeting gene signature (601 genes, Supplementary Table S2). Functional annotation (GO Molecular Function) revealed significant enrichment for snoRNA binding, RNA methyltransferase activity, et al., in the KIRA6-targeting gene signature (Fig. 4h). All these genes were then mapped to their human orthologs. Gene Set Variation Analysis (GSVA) was performed to calculate the enrichment scores of the KIRA6-targeting gene signature. Notably, high enrichment scores of this signature in breast cancer patients correlated with significantly shorter overall survival (HR = 1.509, *P* = 0.0162, Fig. 4i), suggesting that KIRA6 targets a group of genes related to poor prognosis.

### KIRA6 inhibits tumor cell proliferation and induces apoptosis

**Fig. 5:**
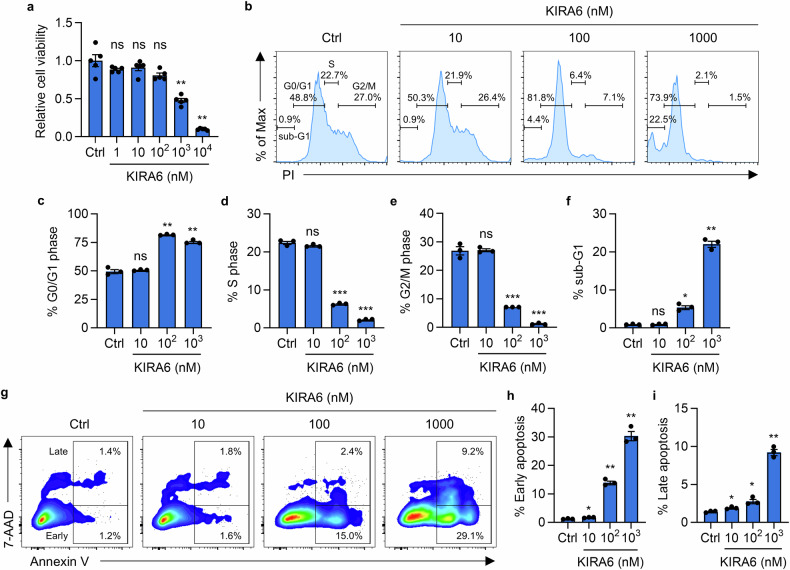
KIRA6 directly inhibits 4T1 tumor cell proliferation by inducing cell cycle arrest and apoptosis. **a** Cell viability of 4T1 tumors cultured for 24 h in control conditions or with KIRA6 treatment at indicated concentrations were measured by CCK-8 assay. *n* = 5; **P < 0.01. **b** Representative flow cytometry analysis for cell cycle distribution of 4T1 cells cultured in indicated conditions. The percentages of G0/G1 phase (**c**), S phase (**d**), G2/M phase (**e**), sub-G1 apoptotic population (**f**) in (**b**) are summarized as mean ± SEM (*n* = 3). *P < 0.05; **P < 0.01; ***P < 0.001. **g** Flow cytometry analysis for apoptosis of 4T1 cells cultured in indicated conditions. The percentages of early-stage apoptosis (**h**) and late-stage apoptosis (**i**) are presented as mean ± SEM (*n* = 3). *P < 0.05; **P < 0.01.

Then, we assessed the direct antitumor effects on 4T1 cells with escalating doses of KIRA6 (1–10,000 nM). KIRA6 significantly suppressed cellular proliferation in a dose-dependent manner as measured by CCK-8 assay (Fig. 5a). In consistence, KIRA6 treatment induced G0/G1 cell cycle arrest, demonstrated by a significant increase in G0/G1 phase cells, concurrent with decreased S phase and G2/M phase populations (Fig. 5b–e). Furthermore, KIRA6 treatment significantly increased the sub-G1 apoptotic population (Fig. 5f). Annexin V/7-AAD staining confirmed KIRA6 treatment significantly induced both early-stage apoptosis and late-stage apoptosis of 4T1 cells (Fig. 5g–i). Collectively, these findings demonstrate that KIRA6 exerts direct antitumor effects through coordinated induction of G0/G1 cell cycle arrest and apoptosis at pharmacologically relevant concentrations.

### KIRA6 attenuates G-CSF production from tumor cells to decrease MDSC generation

**Fig. 6:**
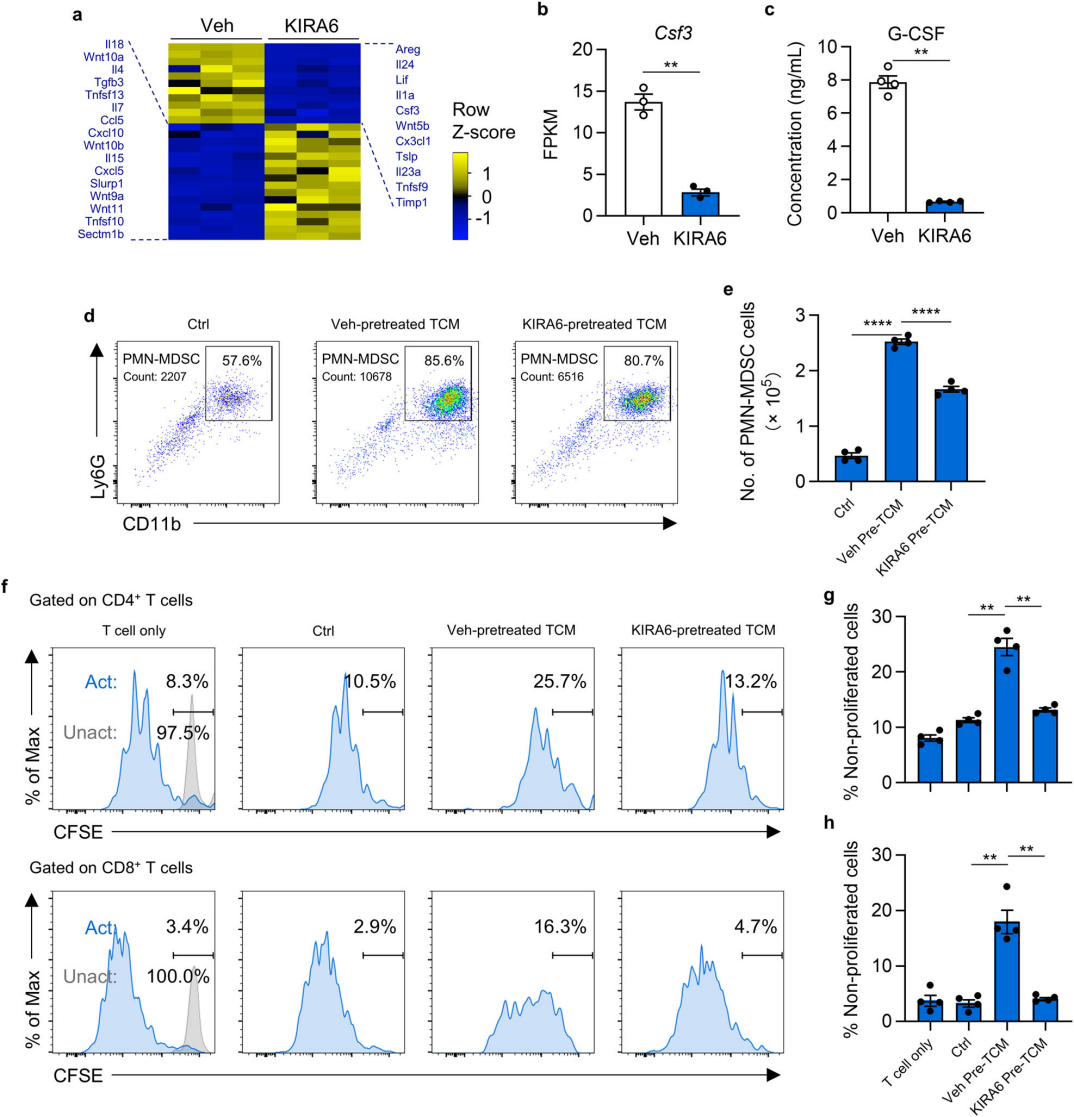
KIRA6 inhibits G-CSF secretion from tumor cells to decrease MDSC generation. **a** Heatmap of differentially expressed cytokines/chemokines. **b** The mRNA expression of Csf3 (G-CSF). The data is shown as mean ± SEM (*n* = 3). **P < 0.01. **c** The secretion of G-CSF in conditioned medium from vehicle or KIRA6 (1 μM) treated 4T1 tumor cells. The results are shown as mean ± SEM (*n* = 4). **P < 0.01. **d** Bone marrow cells were cultured in control medium, TCM from vehicle or KIRA6 (1 μM) pretreated 4T1 tumor cells for 3 days. The number of PMN-MDSC in cultured cells was examined by flow cytometry. **e** The number of PMN-MDSC in (**d**) is summarized as mean ± SEM. n = 4/group; ****P < 0.0001. **f** Splenocytes were cultured alone or co-cultured with MDSC induced with control medium, TCM from vehicle or KIRA6 (1 μM) pretreated 4T1 tumor cells for 3 days (MDSC:T cell = 1:2). The proliferation of T cells was monitored by flow cytometry. The percentage of non-proliferated CD4⁺ T cells (**g**) and CD8⁺ T cells (**h**) is summarized as mean ± SEM (*n* = 4). **P < 0.01.

Cytokines and chemokines are key regulators that promote the production and accumulation of MDSC in tumors. Therefore, we explored the expression of cytokines/chemokines and revealed significant alterations in the expression level of these genes (Fig. 6a). Specifically, the mRNA level of granulocyte colony-stimulating factor (CSF3/G-CSF), a critical factor for generation and mobilization of MDSC, was significantly reduced after being treated with KIRA6 (Fig. 6b). ELISA analysis confirmed the decreased G-CSF secretion (Fig. 6c). To explore the impact on MDSC biology, bone marrow cells were then cultured in conditioned medium from KIRA6-treated 4T1 cells. TCM from KIRA6-pretreated tumor cells exhibited significantly reduced PMN-MDSC induction (Fig. 6d, e). When co-cultured with CFSE-labeled splenocytes, MDSC induced with TCM from KIRA6-pretreated tumor cells failed to suppress proliferation of either CD4⁺ or CD8⁺ T cells (Fig. 6f–h). Thus, KIRA6 remodels the tumor cytokine production to impair the generation and immunosuppressive function of MDSC.

### KIRA6 enhances antitumor immunity and overcomes anti-PD-1 resistance

**Fig. 7:**
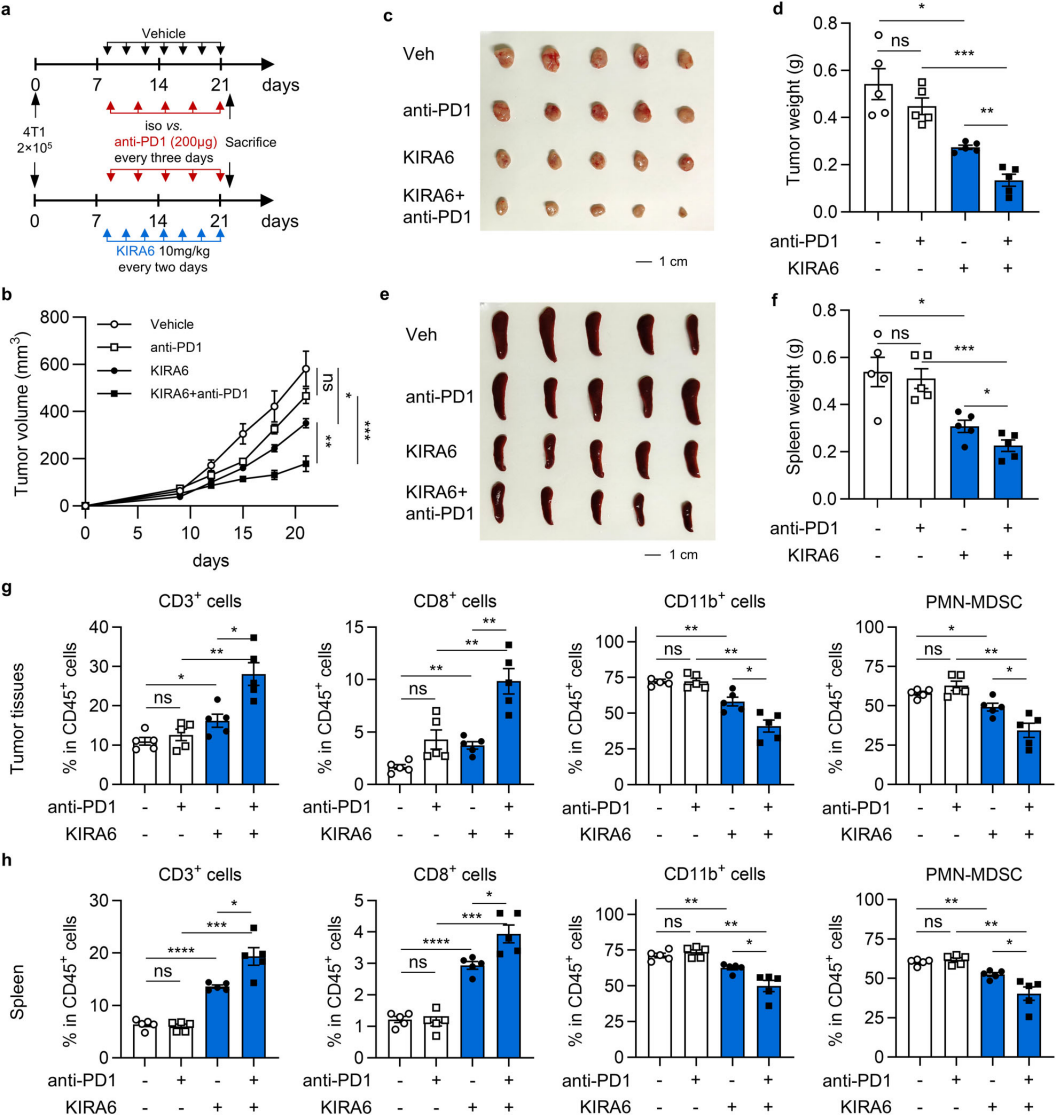
KIRA6 overcomes anti-PD-1 resistance and enhances antitumor immunity. **a** Tumor treatment regimen. 4T1 tumor-bearing mice were treated with vehicle, anti-PD-1 antibody (200 µg *i.p*. every three days, red arrows), KIRA6 (10 mg/kg *i.p*. every two days, blue arrows), or combined treatment from day 9 to 21. **b** Tumor growth curves. Mean tumor volume of 4T1 tumors from the indicated groups is shown as mean±SEM. *n* = 5/group; *P < 0.05; **P < 0.01; ***P < 0.001. **c** Image of tumors from indicated groups at sacrifice. Scale bar, 1 cm. **d** Tumor weight at sacrifice. Results are shown as mean ± SEM. *n* = 5/group; ns not significant; *P < 0.05; **P < 0.01; ***P < 0.001. **e** Image of spleens from indicated groups at sacrifice. Scale bar, 1 cm. **f** Spleen weight at sacrifice. Results are shown as mean ± SEM. *n* = 5/group; *P < 0.05; ***P < 0.001. **g** The percentage of CD3^+^ T cells, CD8^+^ T cells, CD11b^+^ myeloid cells, PMN-MDSC in CD45^+^ cells from 4T1 tumors treated with the indicated drugs was analyzed by flow cytometry. The results are shown as mean ± SEM. *P < 0.05; **P < 0.01. **h** The percentage of splenic CD3^+^ T cells, CD8^+^ T cells, CD11b^+^ myeloid cells, PMN-MDSC in CD45^+^ cells from the indicated groups was quantified by flow cytometry. The data is shown as mean ± SEM. *P < 0.05; **P < 0.01; ***P < 0.001; ****P < 0.0001.

Considering the potent modulation of KIRA6 on both immune microenvironment and tumor cells, we evaluated the therapeutic potential of combining KIRA6 with immune checkpoint blockade. 4T1 tumor-bearing mice were treated with anti-PD-1 (200 µg, *i.p*. every 3 days), KIRA6 (10 mg/kg, *i.p*. every 2 days), or both until sacrifice at day 22 (Fig. 7a). PD-1 blockade failed to inhibit tumor growth in this resistant model. KIRA6 treatment not only significantly suppressed tumor progression alone, but also overcame ICB resistance when combined with anti-PD1 therapy, achieving further tumor suppression (Fig. 7b–d). While anti-PD-1 monotherapy exhibited negligible effect on splenomegaly, the combination of KIRA6 significantly decreased spleen weight (Fig. 7e, f), indicating resolution of tumor-driven extramedullary myelopoiesis.

Moreover, comprehensive immunophenotyping was performed to further analyze immune cell populations in the tumor and spleen. In line with the therapeutic outcome, PD-1 antibody treatment could not significantly modulate the proportion of tumor-infiltrating immune cells. KIRA6 monotherapy increased intratumoral CD3⁺ and CD8⁺ T cell infiltration while decreasing immunosuppressive CD11b⁺ myeloid cells and PMN-MDSC, which were amplified in the combination group (Fig. 7g). In similar, enhanced antitumor immunity, with increased T cell proportions and reduced immunosuppressive myeloid cells, was observed in the spleens from KIRA6 and combo-treated mice (Fig. 7h). These findings suggest that KIRA6 remodels both local and systemic immune landscapes and improves the efficiency of anti-PD-1 therapy.

## Discussion

Immune checkpoint blockade (ICB) therapy is successfully used for cancer treatment in many types of cancers; however, only a small portion of patients benefit from PD-1/PD-L1 blockade therapy because of immune suppression mediated by alternative suppressive immune cells, such as MDSC [8, 10, 41–44]. IRE1α is a key regulator for generation of MDSC, whose kinase activity and RNase activity could be inhibited by KIRA6 simultaneously [39]. However, its potential for targeting MDSC remains unexplored. Here, we evaluated the antitumor activity of KIRA6 for breast cancer, we examined the role of KIRA6 in decreasing MDSC generation, we revealed the effect of KIRA6 on myeloid-promoting cytokine production and survival of tumor cells, and we explored the potential of KIRA6 to improve the benefit from anti-PD-1 therapy. Our work establishes important evidence that KIRA6 potently inhibits systemic and local MDSC in preclinical tumor model and provides a promising agent for overcoming ICB resistance.

MDSC are one of the major immunosuppressive cells that restrain antitumor T cell responses in the TME; therefore, great efforts have been made to develop MDSC-targeting strategies for cancer therapy [17, 18]. Previous studies aimed to abolish MDSC-mediated immune suppression through eliminating MDSC, decreasing MDSC infiltration or abrogating their suppressive functions, such as the use of chemotherapies, chemokine inhibitors, or neutralizing antibodies [17, 23]. These agents effectively abrogate immune suppression of MDSC on T cells and improve antitumor response. However, MDSC rapidly expanded from aberrant myelopoiesis in bone marrow and spleen. We and others have revealed that myeloid progenitor cells significantly increase in the peripheral blood and spleen of cancer patients and serve as an important source of functional MDSC [28, 33, 38]. These observations suggest that tumors systemically regulate myelopoiesis and continuously replenish MDSC through chronic secretion of cytokines, such as G-CSF and GM-CSF. Thus, targeting the source of MDSC is promising for a more sustainable benefit [45]. In this study, KIRA6 treatment significantly suppressed 4T1 tumor growth with decreased MDSC population and enhanced T cell infiltration. Two dosages of KIRA6 treatment potently inhibited MDSC generation and extramedullary hematopoiesis, as indicated by systemically decreased MDSC population and smaller spleen size. Moreover, KIRA6 inhibited MDSC biogenesis and eventually achieved in overcoming resistance to anti-PD-1 therapy. Our work identified KIRA6 as an impressive agent for targeting MDSC generation and overcoming ICB resistance.

The expansion and acquisition of immune suppression capability are orchestrated by several key pathways, offering promising strategies to reprogram MDSC [18]. As a result of high secretory and metabolic activity during the immunosuppressive myelopoiesis in tumor milieu, MDSC are faced with ER stress [34, 35]. When challenged with ER stress, several ER transmembrane sensors mediated signaling cascade determine cell fate, such as IRE1α and PERK [34, 39]. These pathways act as key regulators for generation of suppressive MDSC. IRE1α possesses RNase activities, is activated by trans-autophosphorylation through its own kinase activity [39]. The RNase activity of IRE1α enables processing unspliced XBP1 to its mature production, spliced XBP1 (XBP1s), which encodes the transcriptionally active XBP1 protein [46]. Genetic deletion of IRE1α completely abrogates suppressive activity of PMN-MDSC in tumor model [35]. In this study, pharmacological inhibition of IRE1α with KIRA6 decreased the generation of MDSC and attenuated its immunosuppressive function by downregulating ARG1 expression. On the other hand, cancer cell intrinsic XBP1s favors the synthesis and secretion of cholesterol, which is absorbed by MDSC and drives its immunosuppressive reprogramming [46]. The kinase activity of IRE1α mediates activation of JNK pathway and markedly potentiates the expression of G-CSF and GM-CSF in breast cancer cells [38]. The multiple effects of IRE1α on supporting MDSC generation highlights the potential of inhibiting the kinase and RNase activity simultaneously for cancer immunotherapy. Therefore, KIRA6, other than solo RNase activity inhibitor, such as 4μ8C, MKC8866, or STF-083010 [47, 48], was evaluated for antitumor activity in this study. KIRA6 not only directly decreased MDSC differentiation from bone marrow cells, but also attenuated G-CSF production from tumor cells and therefore blocked the sustainably induction of MDSC. Our data revealed that the dual effect of KIRA6 on blocking MDSC generation. Elucidating the direct binding and inhibition of IRE1α by KIRA6 within MDSCs is valuable for further drug development.

KIRA6 exhibited direct killing on tumor cells in addition to its immune modulation ability. In support with our findings, previous studies have demonstrated IRE1α promotes the survival, growth, and drug resistance of tumor cells, which underline the importance of IRE 1α in tumor biology [49–52]. For example, IRE1α pathway activates c-MYC signaling and promotes prostate cancer [48]; reactivation of IRE1α caused acquired resistance to KRAS inhibitor, and inhibition of IRE1α overcame resistance to KRAS inhibitor [53]; IRE1α RNase silences taxane-induced dsRNA through preventing NLRP3 inflammasome-dependent pyroptosis [54]. We found KIRA6 suppressed cellular proliferation in a dose-dependent manner, and induced cell cycle arrest and apoptosis at sub-micromolar concentration. KIRA6 treatment downregulated the phosphorylation of ERK1/2 and Myc proteins, indicating potent suppression on oncogenic signaling pathways. KIRA6 is also reported to inhibit KIT as well as its downstream signaling modules phosphorylated ERK1/2 at nanomolar concentrations and are sufficient to induce cell death in a KIT signaling-dependent leukemia cell line [55]. This study confirmed the direct cancer-killing effect of KIRA6 on breast cancer at pharmacologically concentrations.

KIRA6 treatment achieved more potent antitumor response and resulted in overcoming ICB resistance when combined with anti-PD1 therapy. The improved T cell response could benefit from various aspects of KIRA6 treatment. On the one hand, KIRA6 systemically reprograms myeloid population, with decreased MDSC in peripheral blood, spleen, and tumor tissue. Spleen is an important source for MDSC generation, while short-term KIRA6 treatment significantly decreased spleen weight, suggesting resolution of extramedullary hematopoiesis. KIRA6 abrogated myeloid cells mediated immune suppression, which is fundamental for overcoming ICB blockade. On the other hand, targeting IRE1α-XBP1 signaling has been found to restore antitumor capacity of T cells in cancer hosts [56, 57]. IRE1α-XBP1 activation is found in T cell from patients with ovarian cancer, and suppresses mitochondrial activity and IFN-γ production. Blocking IRE1α-XBP1 pathway helps to restore the metabolic fitness and antitumor capacity of T cells in cancer hosts [57]. Previous studies suggest that inhibiting IRE1α rejuvenates T cells rather than interfere their antitumor functions. Therefore, KIRA6 abrogated MDSC-mediated immune suppression without impeding T cell functions, making it a promising candidate of small-molecule drug for cancer immunotherapy.

Our study evaluated the antitumor activity of KIRA6 for breast cancer and dissected the underlying mechanism from both MDSC and cancer cell aspect. Our data revealed that KIRA6 potently inhibits MDSC generation and tumor progression in mouse model and uncovered its impressive role in overcoming ICB resistance. However, our study is conducted in animal and cell level. Future preclinical investigations in human models and clinical trials are required for a clearer translational impact.

## Supplementary information

Supplementary Materials-Figure S1 S2 Table S1

Supplementary Materials-Table S2

Supplementary Materials-Original Western Blot

## Data Availability

All data relevant to the study are included in the article or uploaded as supplementary information. The data generated in this study are available within the article and its Supplementary Data files. Any data used in this study that are not included in the paper or supplementary files can be made available upon request from the corresponding author.
