# Supplementary material for: KIRA6 restrains the generation of myeloid-derived suppressor cells and overcomes resistance to anti-PD-1 therapy
Source: Cell Death Dis. 2025 Dec 27;17(1):149. doi: 10.1038/s41419-025-08401-6 (PMC12858800; doi:10.1038/s41419-025-08401-6)
Supplement: Supplementary file 1 — Supplementary Materials-Figure S1 S2 Table S1 [file 41419_2025_8401_MOESM1_ESM.pdf]

Supplementary Materials for

KIRA6 restrains the generation of myeloid-derived suppressor cells and overcomes resistance to anti-PD-1 therapy

Chun Chen[†], Jing Chen[†], Xiaowen Lin[†], Jiali Hu, Yuncong Zhang, Dingjie Liu, Xumei Ouyang, Jing Li, Wenting Li, Shiying Xie, Ya Meng, Meixiao Zhan[*], Yongjun Peng[*], Hong-Wei Sun[*]

† These authors contributed equally to this work

* Corresponding Author

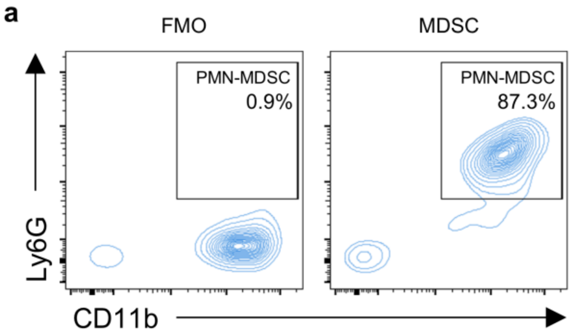

Figure S1. Flow cytometry analysis of MDSC induced from bone marrow cells. (**a**) Bone marrow cells were cultured with 4T1 tumor-conditioned medium (TCM) for 3 days and analyzed by flow cytometry. FMO (Fluorescence Minus One) staining was used for gating of MDSC.

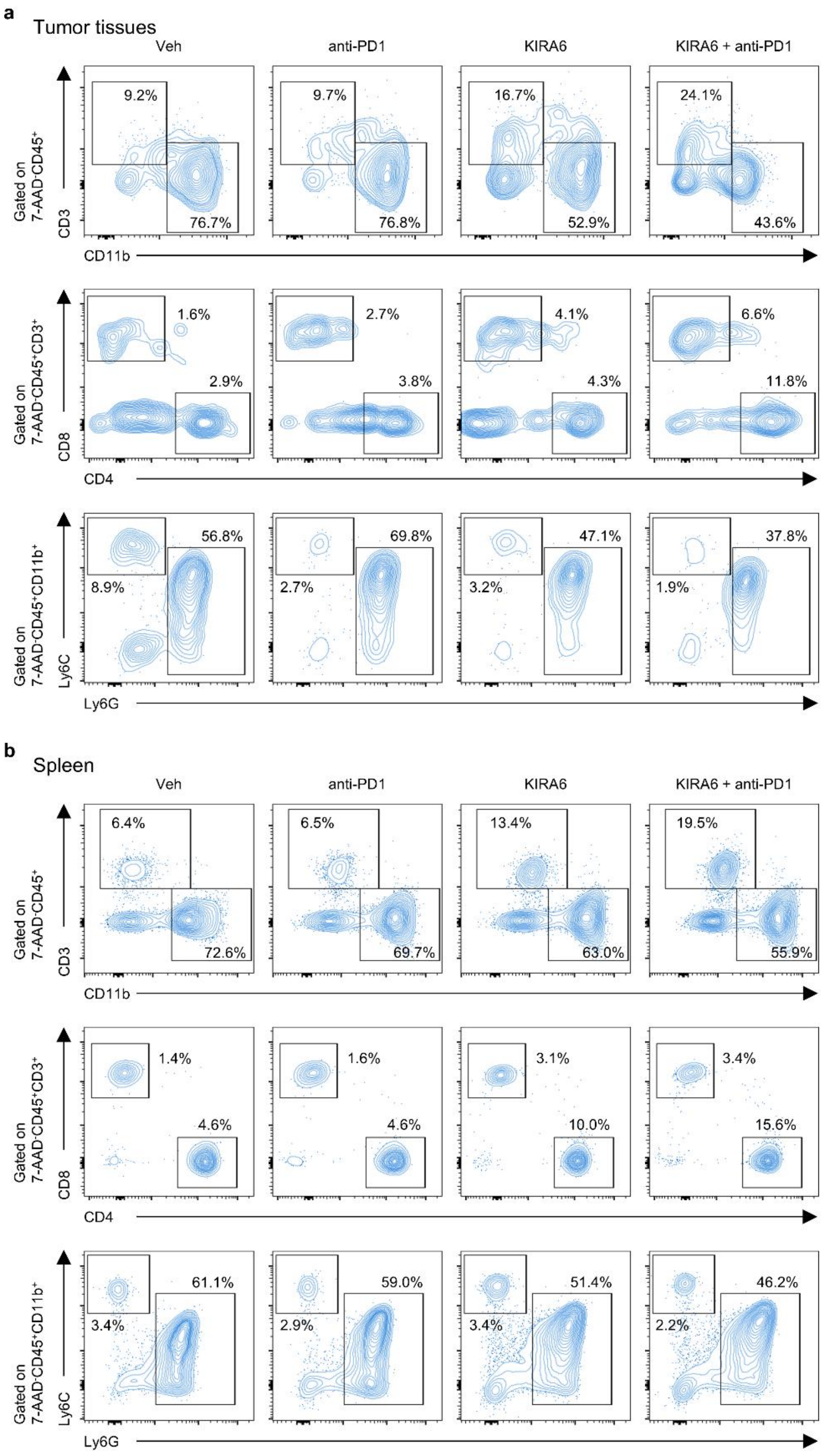

Figure S2. Representative flow cytometry analysis of immune cells from KIRA6 and anti-PD1 treated mice. (**a–b**) The proportion of $CD3^+$ T cells, $CD8^+$ T cells, $CD11b^+$ myeloid cells, PMN-MDSC in $CD45^+$ cells from 4T1 tumors (a) or spleen (b) of mice treated with indicated drugs were analyzed by flow cytometry.

Table S1 Summary of materials

Reagents	Manufacturer	Cat. No.
RPMI1640 medium	Thermo Fisher	C11875500BT
PBS 7.4	Thermo Fisher	C10010500BT
ACK Lysing Buffer	Thermo Fisher	A10492-01
KIRA6	MedChemExpress	HY-19708
Tween 80	MedChemExpress	HY-Y1891
PEG300	MedChemExpress	HY-Y0873
Cell Counting Kit-8	Beyotime Biotechnology	C0039
Brilliant Violet 785™ anti-mouse Ly-6C	BioLegend	128041
Alexa Fluor® 700 anti-mouse CD3	BioLegend	100216
APC anti-mouse/human CD11b	BioLegend	101212
PE/Cyanine7 anti-mouse CD4	BioLegend	100422
PE/Dazzle™ 594 anti-mouse Ly-6G	BioLegend	127648
PE anti-mouse CD8a	BioLegend	100707
7-AAD Viability Staining Solution	BioLegend	420403
APC Annexin V Apoptosis Detection Kit with 7-AAD	BioLegend	640930
APC-Cy™7 Rat Anti-Mouse CD45	BD Biosciences	557659
Invivo anti-mouse PD-1 Recombinant mAb (D265A)	Starter Biotechnology	S0B0594
PAGE Gel Quick Preparation Kit (10%)	Yeasen Biotechnology	20325ES62
BCA Protein Quantification Kit	Yeasen Biotechnology	20201ES86
10×TBST Buffer	Yeasen Biotechnology	60145ES76
Fast Western Blocking Solutions	Yeasen Biotechnology	36122ES76
Primary&Seconfary Antibody Diluent for WB	Yeasen Biotechnology	36206ES76
10×Fast Transfer Buffer	Yeasen Biotechnology	36123ES76
Super ECL Detection Reagent	Yeasen Biotechnology	36208ES76
Universal Electrophoresis and Transfer Buffer (for Western Blot)	Yeasen Biotechnology	20329ES50
Arginase-1 (D4E3M™) XP ® Rabbit mAb	Cell Signaling Technology	93668S
Phospho-ERK1-T202/Y204+ERK2-T185/Y187 Rabbit mAb	Abclonal	AP0974
Phospho-c-Myc-S62 Rabbit mAb	Abclonal	AP0989
ERK1/2 Mouse Monoclonal Antibody	Proteintech	66192-1-Ig
c-MYC Mouse Monoclonal Antibody	Proteintech	67447-1-Ig
β-Actin,Mouse mAb	Yeasen Biotechnology	30101ES60
HRP-conjugated Affinipure Goat Anti-Mouse IgG(H+L)	Proteintech	SA00001-1
Goat pAb to Rb IgG (HRP)	Abcam	ab6721
Mouse G-CSF ELISA Kit	MultiSciences	EK269-96
TRIzol® Reagent	Thermo Fisher	15596018
