# Supplementary figures and images for: KIRA6 restrains the generation of myeloid-derived suppressor cells and overcomes resistance to anti-PD-1 therapy
Source: Cell Death Dis. 2025 Dec 27;17(1):149. doi: 10.1038/s41419-025-08401-6 (PMC12858800; doi:10.1038/s41419-025-08401-6)

**Fig 3d**

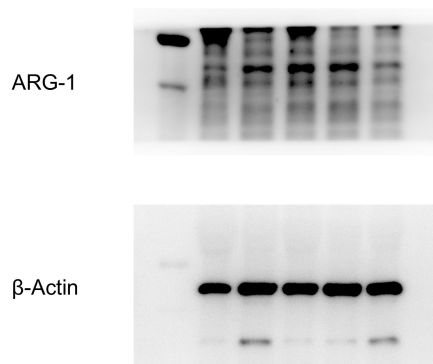

**Fig 6e**

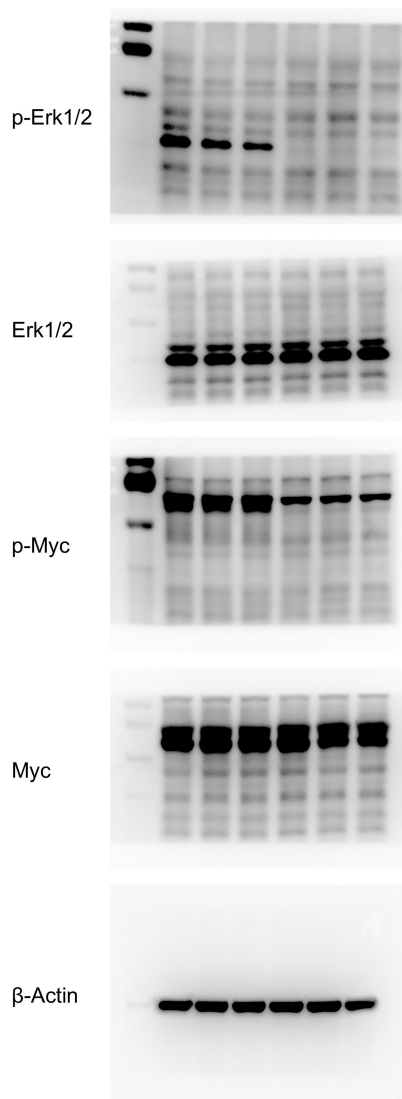

Supplement: Supplementary file 3 — Supplementary Materials-Original Western Blot [file 41419_2025_8401_MOESM3_ESM.pdf]
